# Supplementary material for: Containment of COVID-19: Simulating the impact of different policies and testing capacities for contact tracing, testing, and isolation
Source: PLoS One. 2021 Mar 31;16(3):e0247614. doi: 10.1371/journal.pone.0247614 (PMC8011755; doi:10.1371/journal.pone.0247614)
Supplement: Attachment — Submitted filename: Rebuttal_letter_v3.docx [file pone.0247614.s001.docx]

Dear Dr. Yang,

Thank you, the additional editor and the reviewers for these constructive comments. We have now made every effort to address all major and minor concerns.

In particular, we have run a new series of agent-based simulations to include a stochastic form of transmission, as requested by the additional editor. This new simulation allows a transmitter to reach up to 25 agents in a single simulated day, thus creating the conditions for the simulation of the so-called super-spreaders. This stochasticity in the transmission of the disease is also used to replicate the recently reported data suggesting 80% of secondary infections originate from 10% of infected people (Endo et al., 2020).

The new version of the manuscript now also includes a description of how the chosen variables regulating the disease incidence relate with the reproduction number (R0), showing the settings are consistent with known estimates reported in literature about COVID-19. The manuscript has also been restructured as suggested by one of the reviewers, with the results following the methods section.

Finally, we revised the figures to exclude potentially copyrighted material. The amended figure 1 (previously figure 2) now includes only images that are based on a manipulation of data licensed as CC 4.0 by the European Commission (Global Human Settlement framework).

We hope you will find the revised manuscript appropriate for publication in your journal.

Please see our point-by-point response to the reviewers below.

Sincerely,

Vincenzo Fiore, PhD

Icahn School of Medicine at Mount Sinai – Department of Psychiatry

1 Gustave L. Levy Place, New York, NY 10029-5674

**Additional Editor Comments:

In this manuscript, the authors presented some modeling results about how testing capacity, contact tracing & testing efficacy, disease incidence and proportion of asymptomatic infections jointly determine the spread of COVID-19. The most interesting finding is that, regardless of testing capacity, a low efficacy would lead to continuing growth of the epidemic.
My major concern with this manuscript is the deterministic way for generating transmission dynamics. This is the first time I saw a deterministic (or semi-static) agent-level modeling, although population-level deterministic models have been used widely. The stochasticity of secondary transmissions is the major advantage of agent-based modeling that makes it a better approximation of the real-world epidemics. I don’t think sacrificing this advantage for computational efficiency is justifiable. The number of negative tests were also generated deterministically, with which I’m OK. The magnitude of each epidemic was calibrated to yield desired growth in disease incidence rather than controlled by the more widely accepted threshold parameters such as the basic or effective reproduction number, which also looks questionable to me. We need to know what are the corresponding R0 for the different disease incidence settings, but I don’t see a clear way to establish the link. Even if such a link can be established, we need to be convinced that the corresponding R0 has the threshold property.**

Thank you for these comments and detailed suggestions. In this new version of the manuscript, we now report results obtained in a modified script that allows for stochasticity in the number of daily agents potentially infected by a single transmitter, generating infection clusters and super spreader events.

In the previous version of the simulations, infected agents were: 1) selected randomly as transmitters for the simulated day, in a percentage determined by the incidence settings; and 2) randomly assigned to the category of asymptomatic or symptomatic, determining the number of days they would be allowed to shed the virus, until symptoms onset and isolation. In addition to these existing elements of stochasticity, the new version of the simulation also assigns each agent a value determining the maximum number of infections that each agent can cause, per day. This value varies between 0.01 and 8, and between 0.01 and 25, depending on the simulated condition, allowing the infected agents to transmit the infection to up to 8 or up to 25 healthy agents. Therefore, jointly with the existing initial simulation, that did not allow any variation of this parameter, the new simulations now include one deterministic condition and two stochastic condition of clustered transmission. These have been tested across all the five conditions of contact tracing and testing efficacy, two conditions of percentage of asymptomatic/paucisymptomatic agents, and three conditions of incidence, in a 3x5x2x3 design.

These new simulations allowed us to compute the new testing capacities required for the containment or suppression of viral transmission, highlighting a significant increase in the estimated testing requirements, in comparison with the deterministic condition (Table 1). Importantly, and despite the significant differences in transmission dynamics across conditions, the new simulations confirmed the main finding that, regardless of testing capacity, a low contact tracing and testing efficacy leads to continuing growth of the epidemic (cf. Table 1).

The changes have been described in the amended version of the methods as follows (lines 190-221):

“Once the infected agents were randomly selected, with a probability determined by the incidence setting, they transmitted the infection to a number of contacts that was determined by the condition of clustered transmission. We simulated one deterministic and two stochastic conditions (Figure 2e). In the deterministic condition, each selected infected agent transmitted the virus to one single contact randomly selected among those in the range of travel (Figure 1). Conversely, the stochastic conditions were intended to replicate the suggestion that 80% of secondary transmissions have been caused by 10% of infectious individuals [48]. To this end, all agents were pre-assigned a value at day 1, defining the number of agents that each of them could potentially infect in a single day. These values were determined using an approximation of the hyperbolic function [cluster size=8/x^e ], where a vector x=[1:10] was used to generate 10 cluster size values [8, 1.2, 0.39, 0.17, 0.09, 0.05, 0.04, 0.03, 0.02, 0.01], each assigned with equal probability (10%) to the artificial agents, resulting in a uniform distribution (Figure 2e). The integer part of the cluster size values expressed the number of contacts a single infected agent could infect in one day, whereas the decimal part expressed a probability to reach a further agent. This vector was further modified to simulate the presence of even larger clusters of infections in a second stochastic condition. Here, we divided the top 10% cohort into three sub-cohorts, with values [25, 8.75, 4], respectively associated with probabilities of [1%, 4%, 5%]. These settings resulted in a weighted sum of 8, thus keeping the requirement of 80% secondary transmissions caused by 10% of the infectious agents [48]. For instance, on average, under the larger-cluster stochastic condition, 4 out of 100 agents were assigned a cluster value of 8.75, meaning they would transmit the virus to eight other agents with 100% probability, plus a ninth agent with 75% probability. Likewise, 10 agents were assigned a cluster value of 0.17, meaning they would transmit the virus to only one agent, with 17% probability. To be noted that the average weighted size of the infection clusters for the entire population was equal to 1 across all conditions, thus keeping constant the estimation for the Rt, across cluster size conditions (Figure 3b). Once the cluster size was determined, the targets of the viral transmission were selected at random among the existing agents in the range of travel of the transmitter, as for the deterministic setting. Across all cluster size conditions, if a targeted agent was healthy, it was immediately infected, starting the countdown for symptom manifestation (if any). Conversely, if the contacted agent had been already infected at any point in the past, the propagation of the infection was null. Thus, with the increase in the number of recovered, actively sick or deceased agents, the probability of generating a new infection would decrease, simulating a diminished Rt, due to herd immunity (Figure 3b).”

Finally, in the new version of the manuscript we now also report the reproduction number corresponding to the different disease incidence settings. These values were estimated after the deactivation of the routines set in place to simulate the isolation of agents showing symptoms or found positive to a test and under the conservative assumption that all infected agents could potentially shed the virus for a time similar to the one used for asymptomatic or paucisymptomatic (i.e. up to 8 days after symptoms onset). Under these new conditions, the three incidence levels have been found to be equivalent to effective reproduction values of ~3.05, ~2.18, ~1.31, for the three settings of 35%, 25% and 15%, respectively, in keeping with the estimated values for the SARS-COV2, which has been reported to be approximately between 2 and 3.5 (European Centre for Disease Prevention and Control, 2020c; Flaxman et al., 2020; Riccardo et al., 2020; Zhang et al., 2020; Zhou et al., 2020). This equivalence has been determined by calculating the probability of each infected agent to be selected each day it remains capable of shedding the virus. To be noted that the probability assigned to each agent to transmit the virus to a cluster of healthy agents have been set so to keep the weighted sum constant across cluster size condition, so that the R values here reported do not vary depending on the cluster size setting.

These considerations are now reported in the amended manuscript as follows (lines 174-188):

“The three conditions of incidence simulated different effective reproduction numbers (Rt, or a time-dependent estimate of the basic reproduction, R0 [44]), as they determined the average daily number of secondary infections, putatively varying as a function of the non-pharmacological mitigation strategies set in place [14,24-26]. More precisely, in the absence of a testing and isolation strategy and without the isolation of symptomatic agents at symptom onset [15], the Rt associated with each simulated condition of incidence was estimated using a weighted sum that considered: 1) the probability of each infected agent to be selected each day (i.e., the incidence setting); and 2) the number of days an infected agent could shed the virus (cf. Figure 2c). We established conservative estimates assuming infected agents were allowed to transmit the virus for up to eleven days, i.e., the three days before symptoms onset, plus up to eight days due to either recovery (in mild cases), or hospitalization (in severe cases) [12,13,41]. These settings led to Rt values of ~3.05, ~2.18, ~1.31, for the three settings of 35%, 25% and 15% incidence, respectively. These values are in keeping with current estimations for the COVID-19 [14,41,45-47] and allowed replicating the incremental dynamics in the number of infections reported by several countries (Figure 3a).”

**Specific comments:
1. Page 4, line 83, what shortcomings?**

We have amended the text to clarify the point as follows (lines 84-86)

“Finally, it has not been directly examined whether an increase in testing capacity, albeit necessary, would be in itself sufficient to avoid the large number of undetected infections that have been reported across multiple countries [29-32].”

**2. Page 5, lines 119-120, “environmental conditions” is a misleading phrase which is often interpreted as, e.g., climatic conditions. Maybe “parameter settings”?**

We appreciate your concern. We have amended the text to avoid the misunderstanding, as suggested. The new version of the text now reads (lines 321-324):

“Our first set of simulations covered 60 days across 50 scenarios (i.e., 50 random seeds) and 90 conditions of parameter settings, in a 3 (disease incidence) x 5 (contact tracing and testing efficacy) x 2 (percentage of asymptomatic or paucisymptomatic infections) x 3 (size of infection cluster) design.”

**3. Page 5, lines 130-131. The contact tracing & testing efficacy sounds like “sensitivity” in a broad sense, i.e., combining the success rate of contact tracing and sensitivity of the biological test. You may want to articulate more about its interpretation. I’m confused by “any agent who tested positive” in line 131. Why do you need the infectors to be tested positive? Don’t you assume infected people who are tested negative falsely remain infectious and can transmit?**

That is correct: infected agents that are not found (or tested negative) would keep transmitting the virus until showing symptoms or until recovered. In other words, the infectors had to either show symptoms or test positive to trigger contact tracing. Since all agents showing symptoms are considered tested, so to be included in the computation for the daily testing capacity, the sentence was meant to highlight the fact that contact tracing starts with a positive results of a test.

The simulation of contact tracing and testing assumes that, in any simulated day, the agents who previously tested positive (i.e. tested because they showed symptoms or because they were traced back from another infected agent) become part of a pool of agents to be traced. All the agents in this pool are selected randomly to start the tracing process, until the testing capacity is exhausted, and their contacts are tested, randomly, one by one. For these contacts, the efficacy of contact tracing and testing determines whether the result of the test is positive or not. For instance, agent A shows symptoms, after infecting 40 agents in the previous days. If the efficacy for contact tracing and testing is set to 20%, of the 40 infected agents contacted starting from agent A, only 8 will likely be found positive and the remaining 32 will be left free to transmit the disease.

We have modified the text as follows to clarify the point (lines 229-247):

“To simulate the impact of missed contacts and test sensitivity, which respectively lead to untested infections and false negative tests, five levels of contact tracing and testing efficacy were examined (100%, 80%, 60%, 40% or 20%). This parameter determined the probability that infected agents would be found and correctly tested positive, among those that had been infected by any discovered infected agent, i.e., agents that have showed symptoms or agents that have been traced in a previous day and have tested positive, irrespective of symptom display. Importantly, a missed contact would not only result in leaving one infected agent free to transmit the disease, but it would also leave undetected the entire chain of transmission associated with that missed agent, until it could be traced via a new contact. This simplified mechanism conflates contacts that are missed because they were not tested at all (missed trace) with contacts that were found but then had a false negative test. In real life, a false negative test can have significantly different effects on the behaviour of a positive person in comparison with a test not performed due to a missed trace. However, in the limited settings regulating the simulated behaviour of the agents, the objective was simply to determine the number of agents that were left free to shed the virus. For instance, in a simulated scenario in which there are 100 infected contacts to trace and test, and a contact tracing and testing efficacy of 20%, 80 infected agents will avoid isolation on average. This may be due to a 25% efficacy in contact tracing jointly with 80% test sensitivity (25 contacts are found, 20 tests return positive), or 50% contact tracing efficacy with 40% test sensitivity (50 contacts are found, 20 tests return positive).”

**4. Page 6, lines 166-169. The numbers set for the conditions with growing numbers of infections (i.e., the numbers in dark gray cells of Table 1) are not straightforward to interpret, even after referring to the methods (lines 451-458). Are these numbers practical meaningful or informative?**

We appreciate your concern on this issue. We considered removing those values, since they do not indicate a testing capacity sufficient to curb or suppress viral transmission, but rather a general threshold that would allow testing capacity not to be overwhelmed. However, we ultimately thought these values are still useful to have, at a glance, an idea of the effective reproduction rate, under the different conditions. We have therefore decided to leave this indication, changing the text to clarify the point, as follows (Table legend):

“Simulated testing capacities expressing the availability of tests per thousand agents, per condition. The three values in each cell represent the capacities associated with the three simulated conditions of size of infection cluster (i.e. maximum number of contacts an infected agent can reach in a single simulated day: 1, 8 or 25, respectively). Background colour of an individual cell indicates reported conditions of disease containment: a white background indicates suppression of the transmission (Rt <1), a light grey background indicates containment but not suppression (Rt ≈ 1), and dark grey background indicates exponential growth (Rt > 1). Under conditions characterised by exponential growth, the reported values indicate an approximate threshold that allows the testing capacity not to be exhausted for at least 50 days of simulation time.”

**5. Where is Figure 4?**

We apologise for this mistake: we have missed the upload of figure 4 in the first submission. The figure was subsequently sent to the editor upon request, but it did not reach all reviewers. We have included the figure in the resubmission, and it is now marked as figure 2, due to the restructuring of the manuscript (i.e. the methods are now described before the results).

**6. I’m not sure how meaningful it is to simply embed 100,000 individuals in the three real regions (New York, Southeast Italy and Midlands). The obtained epidemics will almost surely not resemble what truly occurred in these regions. Again, an epidemiologically meaningful way is to calibrate a stochastic or deterministic transmission model to the real disease incidence data in these regions to estimate key parameters such as transmission rate or R0, and then simulate epidemics under hypothetical contact tracing and testing conditions using these key parameters.**

We appreciate your concern on this matter. The purpose of this second set of results is to test whether there are alternative, more effective, ways to perform surveillance testing to aid contact tracing and testing, in comparison with purely randomised tests over the population. In the new version of the manuscript, we now acknowledge the three maps represent examples of three realistic distributions of population density (normally distributed with a single centre, multi-centred or uniform) and therefore they illustrate the differences that can emerge in the effectiveness of the various testing strategies. We have also amended the text to clarify the fact that the results represent a proof of principle indicating that randomised testing strategies should be tailored to the population distribution and general behaviour.

We report these concepts, in the methods, as follows (lines 301-307):

“These regions have been chosen only to illustrate that differences in testing policies can emerge when comparing significantly different population distributions and geographical features. In particular, the three maps differ in presenting: 1) a single high density location with gradually decreasing density as a function of the distance from the centre (New York metropolitan area); 2) a multi-centre organization, with vast areas at very low density or not inhabited (southeast Italy); 3) a completely urbanised region, mostly characterised by uniform low density (Midlands in UK).”

In the Results section (lines 364-367):

“These maps were chosen as representative of significantly different population distributions, and they were used in combination with the two described population-wide travel behaviours, with the purpose of illustrating the effects of different ideal sampling strategies in realistic, albeit simplified, settings.”

In the Discussion section (lines 493-498):

“Although the mixed testing policies we tested in our second set of simulations were developed only for illustrative purposes, they show that uniform random sampling of the population might not always be the optimal strategy to improve containment. Population-level information can instead be used to guide test surveillance strategies, reducing the Rt below 1, even under conditions of low efficacy of contact tracing and testing.”

**7. Page13-14, lines 399-400, as all cases were assumed isolated upon symptom onset, is it meaningful to simulate recovery time or ICU time?**

We agree with you these settings are not strictly useful for the purpose of the proposed study. However, we have decided to report them as they are included in the script. We have modified the text in the manuscript to clarify this point (lines 130-136):

“At present, studies and reports do not yet agree on the relative percentages of the symptomatic infections, due to the differences among regions and countries in the methods for testing and monitoring the infections in the populations and requirements for hospitalization. Therefore, the settings concerning the relative distributions of the symptomatic infections have been included to ease future developments of the script, while for the present simulations, we assumed that all symptomatic infections entered isolation the day they displayed symptoms.”

**8. Page 14, lines 414-428, once an infectious person was chosen to generate a transmission, will this person be eligible again for future selection of transmitters?**

Any agent who has not been isolated (e.g. because tested positive or after symptom onset) or fully recovered (in the case of asymptomatic/paucisymptomatic) can be selected as a transmitter. This does happen multiple times, especially under the condition of high incidence, due to the increased probability of being selected. We have amended the text to clarify this point, as follows (lines 170-174)

“To this end, the transmission of the virus was simulated starting from the daily number of infected, non-isolated agents who were within 3 days of displaying symptoms [12,16,42,43], and randomly selecting a percentage of these (15%, 25% or 35%, depending on the simulated condition of incidence), irrespective of whether they had been already selected at any prior point in time.”

**9. Page 15, line 432, “These were randomly selected”, how is the amount of such selection of cases for contact tracing determined? Does it depend on assumed testing capacity?**

That is correct: the testing capacity determines the number of infected agents that will be traced. We have amended the text to make this point explicit (lines 225-229)

“These were randomly selected one by one, removing them from future pools and adding one positive test to the count of the day. Then, the agent under examination was used to trace the agent (if any) that had been the origin of her infection and the agents (if any) that she had infected at any time during the simulation, until exhaustion of the testing capacity.”

**10. Page 15, line 449, The ratio of untested infected over healthy agents should be most below 1, how can it be equal to the number of negative tests? Why is it necessary to set a maximum (20) for the number of negative tests per each positive one? Is this maximum number supported by any reference?**

Thank you for pointing this out. We needed to configure the simulations so to include a number of negative tests for each positive one, so it seemed plausible to assume that under a condition in which there are many (non-isolated) infected agents and few healthy agents remaining, the rate of positivity would be very high. The opposite would be true under the opposite conditions, where a large number of healthy agents is present and the number of non-isolated infections is low. This consideration led to the idea of a number of negative tests based on the relative ratio among the two groups, i.e. a number of negative tests to be added for each positive, equivalent to the number of healthy agent present per each infected -non isolated- agent. However, such ratio would have led to exceedingly high numbers of negative tests in the early days of the simulations (~50 infections at day one over the entire population), so we added a threshold. The threshold was established so that the testing capacities resulting in containment would also account for a positivity rate below 5%, as by indication of the World Health Organization (cf. Public health criteria to adjust public health and social measures in the context of COVID-19; 12^th^ May 2020).

We have amended the text in the manuscript to clarify this point, as follows (lines 252-261):

“For each positive test found with contact tracing, the simulations added a number of negative tests, which contributed to reach the daily maximum testing capacity. This was equivalent to the number of healthy agents per active (non-isolated) infected agent, updated daily, up to a maximum of 20 negative tests per each positive one. For instance, under a scenario in which 50 thousand healthy agents and 5 thousand infected non-isolated agents are present in the same day, the number of negative tests added for each positive would be of 10. This mechanism was implemented so that the optimal testing capacities associated with Rt≤1 would always be sufficient to account for a positivity rate below 5%. This threshold was set on May 12th 2020 by the World Health Organization as a key requirement to lift or avoid lockdowns [7].”

**11. Page 16, lines 465-467, how is ‘concentration” calculated? How do you define “latest outbreak”?**

We have clarified these terms in the new version of the manuscript. We defined the “latest outbreak” as the area in the map with the highest concentration of new known infections reported the day prior to the testing. The highest concentration was itself determined by defining a cell (i.e. a squared portion of the map) centred on each new reported infections and assigning a value to each of these cells, depending on the number of new infections included within the borders of these cells. The new text describes this procedure as follows (lines 277-284)

“To flexibly target the areas in the map reporting the latest outbreaks of new infections, the cells were centred on the coordinates of highest concentration of new infections, as recorded the day prior to the sampling. To determine these coordinates, we defined a cell centred on each new infection recorded the day prior to the sampling and assigned each of them a value equivalent to the number of new known infections (i.e. due to positive test or symptom onset) included in the cell. The coordinates associated with the highest value would determine the highest concentration of new infections. In case of multiple cells reporting the same number of new infections, a cell would be selected randomly.”

**12. Figure 3, many colored curves cannot be seen clearly. Can you use smaller symptoms and thinner lines? If some settings do not really differ much from each other, you may consider dropping some settings.**

We appreciate your concern on this matter. Figure 5 (in the previous version of the manuscript, figure 3) is used to illustrate how different policies may overlap, or diverge significantly, depending on the tested conditions of population density and distribution (i.e. the probabilistic maps) and travel behaviour. As discussed in the answer to question #6, this second set of results is meant to show that, when planning surveillance testing: 1) randomised tests might not be the most efficient option; 2) alternative policies should be tailored considering geography and travel behaviour of the population. We do not claim the policies we tested in particular should be used, as they are based on important simplifications and assumptions that make them only useful as an illustrative example. In this sense, we think it is important to display across all panels the 5 best performing policies (plus the reference of contact tracing and testing alone and aided by random sampling) to give the reader a picture at a glance of this overlapping and divergence across scenarios.

**Reviewer #1: Using simulation models, this article demonstrates the importance of contact tracing and testing (especially the former) in controlling the COVID-19 epidemic, and I recommend that it should be published with minor revisions. The manuscript appears technically sound, and the data support the conclusions.
I suggest the overall format of the article be revised, as the results are currently presented before the methods. It would be more conventional and easier to understand if the methods are presented first. Some figures that are referred to in the text were not included in the manuscript, which was a bit confusing.**

We thank you for these encouraging comments and we appreciate the suggestion to modify the order of the main sections of the manuscript to facilitate the understanding of its content. The new version of the manuscript presents the methods before the results, in a conventional way.

We also apologise for the missing figure 4 in the first submission. We have included this figure in the resubmission, and it is now referenced as figure 2, due to the changes in the structure of the manuscript.

**I have a few suggestions for minor revisions:
1. Line 89-115: these 2 paragraphs don’t really fit into the introduction, as they include a mixture of research methods and results. It would be easier to read the article if all the methods are first presented separately (in a methods section), and then the results presented. Eg. Line 92-102 and 108-111 describe methods; 103-104 and 112-115 describe results. All the “see methods” comments in the results section can be removed if the results are presented after the methods.**

Thank you for these suggestions. As mentioned above, in the amended manuscript, the methods section now precedes the Results and Discussion sections, as you suggested. We have also modified the Introduction section to exclude the reference to the results and simplify the description of the experimental design. We acknowledge this description could be limited in the Methods section, but considering the presence of 90 conditions over four dimensions, we think it may facilitate the reader to be introduced to this information early in the manuscript.

The final paragraphs of the Introduction now read as follows (lines 95-113):

“In the first set of simulations, we estimated the testing capacity that would allow for identification and isolation of a number of infections sufficient to break the chain of transmission of SARS-CoV-2. We tested the sensitivity of this measure along four dimensions, in a 3 (disease incidence) x 5 (contact tracing and testing efficacy) x 2 (percentage of asymptomatic/paucisymptomatic infections) x 3 (size of infection clusters) condition design. Specifically, three different levels of disease incidence values determined the increment of new daily infections (e.g., due to different non-pharmacological mitigation policies in place [14,24]). Five different rates of contact tracing and testing efficacy determined the number of infected subjects that were found or missed by the testing process (e.g., due to false negatives or untraced contacts [17,29]). Two estimates to determine the percentage of asymptomatic or paucisymptomatic infections [18,19,35], which were used to define the percentage of the infected population that would not self-isolate after contracting the disease. Finally, three conditions of clustered infection transmission were used to allow each infected agent to transmit the virus up to 1, 8 or 25 healthy agents, depending on the condition.

In the second set of simulations, we focused on the hypothesis that population-level analysis of geographical distribution and travel behaviour could be used to improve randomised surveillance testing methods [3,4,36], so aiding the contact tracing process in finding those infected agents that were left undetected.”

**2. Line 100 should read “due to false negatives”**
**Line 307 should read “irrespective of symptoms”
Line 397-400: The number of days required to develop symptoms, if any (Figure 4b), the number of days to reach full recovery, with a bimodal distribution due to the shorter time of recovery for the asymptomatic (Figure 4c), and the time spent in intensive care units (Figure 4d) were also predetermined). I suggest these technical details be expanded and clarified: what exactly were the predetermined numbers that were used in the models for the incubation period, recovery period and intensive care unit period? Also, the figures referred to were not included in the manuscript, so I cannot comment on them (Figure 4a-d are missing from the manuscript).**

Thank you for pointing out these mistakes. We have corrected them in the amended version of the manuscript.

We apologise for the missing figure. We have included it in the resubmission and it is now numbered as figure 2.

Finally, in the new version of the manuscript we now report the details about the various distributions used for the settings regulating: the number of days prior to symptoms onset, the number of days required to reach recovery or to be spent in the ICU, as follows (lines 138-147):

“The second value of 30% was considered as a plausible alternative [19], so as to explore variations in testing capacities and analyse the consistency of the effects determined by the variations in contact testing efficacy (cf. Table 1). The number of days required to develop symptoms, if any (rounded Pearson distribution: μ= 5.5; σ = 2; skewness = .6; kurtosis = 3; Figure 2b), the number of days to reach full recovery, with a bimodal distribution due to the shorter time of recovery for the asymptomatic or paucisymptomatic [12,13] (μ= 21; σ = 5; skewness = 0; kurtosis = 3, for all agents, divided by four for the subclinical cases, so to reach recovery within 8 days in ~99% of the cases; Figure 2c), and the time spent in intensive care units (μ= 5; σ = 1.5; skewness = 0; kurtosis = 3; Figure 2d) were also predetermined [39-41].”

**Reviewer #2: This manuscript discusses potential methods to contain the COVID-19 through the multi-agent simulations. This research is really interesting, and the authors explained their goals, simulation methods, and the results comprehensively. Here are my comments:**

Thank you for this highly encouraging comment. We have now worked on the manuscript to address all the concerns and improve the clarity of our study.

**1) The whole manuscript needs review and edition in terms of English proficiency. Examples are lines 46-53.**

Thank you for pointing this out. The manuscript has been reviewed carefully by the authors to improve the language.

**2) Lines 55-58 explain some suggestions made by WHO, but there is no reference to the WHO link for these suggestions.**

Thank you for this comment. In the new version of the manuscript we have now added a reference to a report released by the WHO in May, by the title “Public health criteria to adjust public health and social measures in the context of COVID-19”, which details the methods for public health surveillance and the identification of infections and their contacts (line 60 in the amended version of the manuscript).

**3) In lines 89-90, the authors declared that they focused on pre-symptomatic and asymptomatic cases. They also assumed symptomatic patients would not transfer the disease. This assumption is a little strict and may not be satisfied in reality. Some symptomatic patients may still transmit the disease unless they confirmed positive. It is suitable if the authors explain why they removed symptomatic cases from possible transmission sources or run the simulation by considering this group of patients as a potential infection source.**

We appreciate your concern on this issue. At the time we established the key settings for the simulations, it was necessary to define how long each infected agent would be able to shed the virus. This question in turn generated other questions that could not be answered, as the percentage of severe cases and the distribution of the number of days prior to hospitalization (and presumably isolation) was not clearly reported in literature. Similarly, mild cases could potentially transmit the disease for several days after developing symptoms, however this time interval is also affected by several environmental factors. For instance, an infected person with mild symptoms may not have access to a test facility, they might refuse to take a test (e.g. due to costs, social norms, political ideology etc. cf. Rubin 2020 First It Was Masks; Now Some Refuse Testing for SARS-CoV-2; JAMA. doi:10.1001/jama.2020.22003), or they may decide to not self-isolate even when knowingly positive (e.g. if they do not have sick leave). In the absence of these details, we decided to simplify the simulation settings and we assumed that all agents developing symptoms would be immediately tested and would be able to either self-isolate or be isolated in a healthcare facility. Importantly, this choice allowed us to focus our findings on asymptomatic and pre-symptomatic transmission, which is playing a major role on the COVID-19 pandemic (e.g. see the recent Scientific Brief released by the CDC: Community Use of Cloth Masks to Control the Spread of SARS-CoV-2, https://www.cdc.gov/coronavirus/2019-ncov/more/masking-science-sars-cov2.html).

We also acknowledge that this is an important limitation of the study, however we propose that variations in the results associated with an increase in agents who might not isolate immediately at symptom onsets can be inferred by comparing the two settings (20% and 30%) for the number of asymptomatic/paucisymptomatic. More precisely, a less stringent rule for symptomatic infections would lead to an increase in terms of testing capacity requirements, but low levels of contact tracing and testing efficacy would still be associated with exponential growth, irrespective of the testing capacity. These considerations have been included in the Discussion section as part of the limitations of the study, as follows (lines 518-532):

“Concerning the third simplification and the immediate isolation of symptomatic infections, we acknowledge that this assumption implies that agents displaying symptoms would have immediate access to a test and would be able to either self-isolate or be isolated in a healthcare facility. While this choice allowed us to avoid further clinical assumptions (e.g., relative percentage of severe and mild infections, or time prior to hospitalization, when necessary), as well as environment specific settings (e.g., access to sick leave, willingness to comply with social norms, political ideology, etc.), it focussed our investigation on the pre-symptomatic and asymptomatic/paucisymptomatic transmission, which plays a fundamental role in the COVID-19 pandemic [17,18,22]. Furthermore, since the cohort of the asymptomatic/paucisymptomatic infections is only isolated after a positive test, we can infer that a less stringent isolation rule for the symptomatic infections would lead to similar results as those reported in association with an increase in the percentage of the non-isolating cohort (from 20% to 30%, cf. Table 1). Therefore, we would expect to find an increase in terms of the contact tracing and testing resources, when containment is possible, and exponential growth under conditions of low contact tracing and testing efficacy, irrespective of the testing capacity.”

**4) Why two factors, contact tracing, and testing efficacy, are considered together? Especially, in lines 440-441, how they calculated 20% of contact tracing and testing efficacy might result from 25% of tracing with 80% test reliability. It would be better if they could provide more details about this strategy.**
We acknowledge this settings conflates two distinct phenomena that in real life conditions should be kept separate. In real life, those who received a false negative test feel reassured and may behave in a significantly different way than if they had not been contacted at all for a test. However, in the limited setting of our simulations, we do not have ways to simulate different types of behavioural responses to the news related to a test performed (e.g. a change towards a risk prone behaviour) and the psychological effect of the false negative test is lost. Under these simplified conditions, the difference between a missed contact due to inefficient contact tracing or a false positive due to reduced test sensitivity becomes negligible. We have clarified this issue in the amended text as follows (lines 237-247):

“This simplified mechanism conflates contacts that are missed because they were not tested at all (missed trace) with contacts that were found but then had a false negative test. In real life, a false negative test can have significantly different effects on the behaviour of a positive person in comparison with a test not performed due to a missed trace. However, in the limited settings regulating the simulated behaviour of the agents, the objective was simply to determine the number of agents that were left free to shed the virus. For instance, in a simulated scenario in which there are 100 infected contacts to trace and test, and a contact tracing and testing efficacy of 20%, 80 infected agents will avoid isolation on average. This may be due to a 25% efficacy in contact tracing jointly with 80% test sensitivity (25 contacts are found, 20 tests return positive), or 50% contact tracing efficacy with 40% test sensitivity (50 contacts are found, 20 tests return positive).”

**5) Lines 136-137: "... would not show symptoms severe enough to induce... . Therefore in our simulations, these asymptomatic agents ... ". A symptomatic ( no matter mild or severe )patient may not be included in the asymptomatic category. Suppose the authors want to have these types of patients in the asymptomatic category. In that case, it is suitable they define their asymptomatic group and explain that this group contains both patients with no symptoms or those who do not show symptoms severe enough to induce self-isolation or hospitalization.**
Thank you for pointing this out. We have amended the text so that the cohort of agents who do not isolate at symptoms onset is explicitly described as composed by both the asymptomatic and the paucisymptomatic infections.

**6) line 145: Define effective reproduction.**
We have amended the manuscript to define the effective reproduction number and present an estimation of these values associated with the chosen three levels of disease incidence. The text has been amended as follows (lines 174-188):

“The three conditions of incidence simulated different effective reproduction numbers (Rt, or a time-dependent estimate of the basic reproduction, R0 [44]), as they determined the average daily number of secondary infections, putatively varying as a function of the non-pharmacological mitigation strategies set in place [14,24-26]. More precisely, in the absence of a testing and isolation strategy and without the isolation of symptomatic agents at symptom onset [15], the Rt associated with each simulated condition of incidence was estimated using a weighted sum that considered: 1) the probability of each infected agent to be selected each day (i.e., the incidence setting); and 2) the number of days an infected agent could shed the virus (cf. Figure 2c). We established conservative estimates assuming infected agents were allowed to transmit the virus for up to eleven days, i.e., the three days before symptoms onset, plus up to eight days due to either recovery (in mild cases), or hospitalization (in severe cases) [12,13,41]. These settings led to Rt values of ~3.05, ~2.18, ~1.31, for the three settings of 35%, 25% and 15% incidence, respectively. These values are in keeping with current estimations for the COVID-19 [14,41,45-47] and allowed replicating the incremental dynamics in the number of infections reported by several countries (Figure 3a).”

**7) Review lines 172-176.**
We have carefully revised the entire text. These lines in particular have been also moved in the Discussion section, as we assessed they present an interpretation of our findings, rather than a description of the results.

**8 ) In Figure 1, Is the contact tracing efficacy means contact tracing and testing efficacy? If yes, please correct it. If not, where the authors include testing efficacy in this figure?**
We have amended the figure as suggested: in the amended version of the manuscript this is now figure 4. Thank you for pointing this out.

**9) Lines 279-281: The authors claim in low contact tracing and testing efficacy, the number of daily infections keeps growing regardless of the testing capacity. In Figure 1, the number of daily infections does not have increasing trend for low incidence even in low levels of contact tracing and testing efficacy( If we assume the authors means contact tracing and testing efficacy instead of contact tracing efficacy). Moreover, 40% of contact tracing and testing efficacy is enough to contain a disease with medium incidence.**
We agree that those lines did not adequately summarise our findings. Our results indicate that, given a sufficient testing capacity, contact tracing and testing efficacy may lead one of three alternative outcomes: suppression, containment, or exponential growth. In the first case, no pre-symptomatic or asymptomatic infection is present by day 60 of the simulated time (cells with white background in Table 1). In the second case, the simulations show a number of pre-symptomatic or asymptomatic infections still present at day 60, but this value is equal or less the value recorded at day 1 (cells with light grey background in Table 1). In the third case exponential growth is not avoided, irrespective of testing capacity (cells with dark grey background in Table 1). The conditions highlighted in this question –15% incidence, jointly with 40% and 20% contact tracing and testing efficacy, Figure 4a (previously numbered as Figure 1a) and 25% incidence, jointly with 40% efficacy, Figure 4c (previously Figure 1c) – belong to the second case of containment without suppression.

We have amended the text to correct the mistake(lines 446-449):

“First, we found that at low levels of contact tracing and testing efficacy, the Rt of the infection remains equal to or above 1, indicating the disease is not suppressed and under some conditions is not even contained, irrespective of the testing capacity.”

**10 ) In lines 293-296: The authors explain that "beyond the 60% threshold of contact tracing and testing efficacy, there is a steady decline in the numbers of infection across all simulated levels of incidence and across all levels of asymptomatic infections." Still, there is no figure or table to support this explanation for the asymptomatic level of 30%. Figure 1 shows the results only for the asymptomatic level of 20%.**
You are correct in pointing out the results associated with the condition of 30% asymptomatic/paucisymptomatic is not reported in a separate figure. We decided to avoid presenting images that would only show a significant difference in the testing capacity values. For the same reason in this new version of the manuscript we do not report as a separate figure the dynamics associated with the different infection cluster sizes. However, table 1 does report all the data relative to the different conditions, including condition-specific testing capacity and the division into three sets of results (see the answer to question #9).

We report the table 1 below for ease of read:

| Table 1. Estimated testing capacities per simulated condition.  Simulated testing capacities expressing the availability of tests per thousand agents, per condition. The three values in each cell represent the capacities associated with the three simulated conditions of size of infection cluster (i.e. maximum number of contacts an infected agent can reach in a single simulated day: 1, 8 or 25, respectively). Background colour of an individual cell indicates reported conditions of disease containment: a white background indicates suppression of the transmission (R_t_ <1), a light grey background indicates containment but not suppression (R_t_ ≈ 1), and dark grey background indicates exponential growth (R_t_ > 1). Under conditions characterised by exponential growth, the reported values indicate an approximate threshold that allows the testing capacity not to be exhausted for at least 50 days of simulation time. | | | | |
| --- | --- | --- | --- | --- |
|  |  | **Incidence: 15%** | **Incidence: 25%** | **Incidence: 35%** |
| Asymptomatic: 20% | 100% contacts traced | 0.7 - 0.7 - 0.7 | 1.7 - 2.4 - 2.6 | 3.6 - 5.5 - 6.8 |
|  | 80% contacts traced | 0.7 - 0.7 - 0.7 | 1.7 - 2.4 - 3.0 | 3.6 - 5.5 - 7.1 |
|  | 60% contacts traced | 0.7 - 0.7 - 0.7 | 1.7 - 2.9 - 3.4 | 4.5 - 8.2 - 9.2 |
|  | 40% contacts traced | 0.7 - 0.7 - 0.7 | 2.0 - 2.9 - 3.4 | 15 - 20 - 25 |
|  | 20% contacts traced | 0.7 - 0.7 - 0.7 | 3.0 - 3.2 - 3.4 | 30 - 35 - 40 |
| Asymptomatic: 30% | 100% contacts traced | 0.8 - 0.8 - 1.0 | 2.0 - 3.2 - 3.9 | 4.0 - 6.8 - 7.9 |
|  | 80% contacts traced | 0.8 - 0.9 - 1.3 | 2.0 - 3.7 - 4.0 | 4.0 - 6.8 - 9.1 |
|  | 60% contacts traced | 0.8 - 1.0 - 1.3 | 2.0 - 3.8 - 6.8 | 6.0 - 9.8 - 11.9 |
|  | 40% contacts traced | 0.8 - 1.0 - 1.3 | 2.3 - 3.8 - 6.8 | 35 - 37 - 40 |
|  | 20% contacts traced | 0.8 - 1.0 - 1.3 | 3.3 - 4.0 - 6.8 | 50 – 52 - 55 |

The legend of Figure 4 (previously, Figure 1) has been changed as follows to clarify this issue (lines 766-768):

“These simulations illustrate the dynamics found across all conditions of symptomatology in the population distribution and across all conditions of infection cluster size. For these simulations, the percentage of asymptomatic/paucisymptomatic infections is fixed at 20% and the size of the infection cluster is fixed to 1 across all agents (i.e., deterministic setting).”

**11) Lines 351: Please explain what "we simulated the reproduction of the virus in terms of ..." means.**
Thank you for pointing this out. In that part of the Discussion section we presented the limitation of relying only on deterministic viral transmission in the simulations. This part of the Discussion section has been deleted in the new version of the manuscript as it was no longer necessary. In the amended version of the manuscript, we now report also new results obtained including two separate conditions of stochastic viral transmission in which we controlled the dimension of the infection cluster and allowed single agents to transmit the virus to up to 25 contacts, per day.

**12) Please add Figure 4 to the pdf file**
Our apologies for this mistake. The figure was inadvertently excluded from the initial submission, and it has now been included in the resubmission, as Figure 2, due to the restructure of the text (Methods before the Results).

**13) Line 402: Please explain the "range of virus transmission."**
We agree with you this detail required further clarifications. The simplified travel behaviour assigned to the artificial agents determines the boundaries of their movement, and therefore it also determines which agents they can establish a contact with and potentially infect. We have amended the text to clarify this issue, as follows (lines 149-157):

“Finally, each agent was assigned to one of three possible “travel cohorts”, defining the range of movement of each agent, and therefore their range of viral shedding: the entire map (1000x1000 pixels, comprising an entire region or metropolitan area), a medium size sector (300x300 pixels, e.g. comprising two boroughs or two separate towns, depending on the map) or a small size sector (100x100 pixels, e.g. within one city or one borough, cf. Figure 1). We simulated two conditions in terms of cohort distribution for the large, medium and small sector travel behaviour, as follows: 5%-5%-90% (skewed distribution) or 30%-30%-40% (uniform distribution). These simplified settings have been chosen only to illustrate the effects the different policies of surveillance testing have under significantly different population-wise behaviours.”

**14) Line 423: Why the authors supposed each contact will result in infection with probability one when the contract is between an infector and a susceptible person? Some contacts may not yield an infection.**

We agree with you that this was an important limitation in the previous version of our study. In the new version we have addressed this problem by including a further dimension in the study design in which we controlled the size of the cluster of infections that could potentially be created by a single infected agent. In the new settings, a few agents could potentially generate up to 25 infections, but at the same time several agents had low chances to generate any infection at all. The details of this mechanism are described in the amended version of the methods as follows (lines 190-221):

“Once the infected agents were randomly selected, with a probability determined by the incidence setting, they transmitted the infection to a number of contacts that was determined by the condition of clustered transmission. We simulated one deterministic and two stochastic conditions (Figure 2e). In the deterministic condition, each selected infected agent transmitted the virus to one single contact randomly selected among those in the range of travel (Figure 1). Conversely, the stochastic conditions were intended to replicate the suggestion that 80% of secondary transmissions have been caused by 10% of infectious individuals [48]. To this end, all agents were pre-assigned a value at day 1, defining the number of agents that each of them could potentially infect in a single day. These values were determined using an approximation of the hyperbolic function [cluster size=8/x^e ], where a vector x=[1:10] was used to generate 10 cluster size values [8, 1.2, 0.39, 0.17, 0.09, 0.05, 0.04, 0.03, 0.02, 0.01], each assigned with equal probability (10%) to the artificial agents, resulting in a uniform distribution (Figure 2e). The integer part of the cluster size values expressed the number of contacts a single infected agent could infect in one day, whereas the decimal part expressed a probability to reach a further agent. This vector was further modified to simulate the presence of even larger clusters of infections in a second stochastic condition. Here, we divided the top 10% cohort into three sub-cohorts, with values [25, 8.75, 4], respectively associated with probabilities of [1%, 4%, 5%]. These settings resulted in a weighted sum of 8, thus keeping the requirement of 80% secondary transmissions caused by 10% of the infectious agents [48]. For instance, on average, under the larger-cluster stochastic condition, 4 out of 100 agents were assigned a cluster value of 8.75, meaning they would transmit the virus to eight other agents with 100% probability, plus a ninth agent with 75% probability. Likewise, 10 agents were assigned a cluster value of 0.17, meaning they would transmit the virus to only one agent, with 17% probability. To be noted that the average weighted size of the infection clusters for the entire population was equal to 1 across all conditions, thus keeping constant the estimation for the Rt, across cluster size conditions (Figure 3b). Once the cluster size was determined, the targets of the viral transmission were selected at random among the existing agents in the range of travel of the transmitter, as for the deterministic setting. Across all cluster size conditions, if a targeted agent was healthy, it was immediately infected, starting the countdown for symptom manifestation (if any). Conversely, if the contacted agent had been already infected at any point in the past, the propagation of the infection was null. Thus, with the increase in the number of recovered, actively sick or deceased agents, the probability of generating a new infection would decrease, simulating a diminished Rt, due to herd immunity (Figure 3b).”

**15) Please explain why the null propagation of infection (Line 425) simulates a diminished effective reproduction(number)?**
As the number of agents who can no longer be infected increases, because they are immune, sick, or deceased, the probability to generate a new infection decreases. This is due to the fact that the targets of infections are always selected randomly within the range of travel of each single agent. This effect is now illustrated in the added Figure 3b, which displays a series of simulation in which contact tracing and testing as well as self-isolation at symptom onset have been deactivated. We have modified the text to clarify this point, as follows (lines 216-221):

“Across all cluster size conditions, if a targeted agent was healthy, it was immediately infected, starting the countdown for symptom manifestation (if any). Conversely, if the contacted agent had been already infected at any point in the past, the propagation of the infection was null. Thus, with the increase in the number of recovered, actively sick or deceased agents, the probability of generating a new infection would decrease, simulating a diminished Rt, due to herd immunity (Figure 3b).”

In the legend of Figure 3, we report (lines 748-756):

“In the bottom panel (b) the error bands (mean and standard deviation) illustrate on a logarithmic scale the simulated number of total infected agent in a population of 100’000 agents, varying condition of incidence and size of the infection clusters. These simulated scenarios assumed no testing and isolation strategy was in place and symptomatic agents were not automatically isolated at symptom onset. The resulting exponential increase in the number of infections replicated the dynamics described for the real case scenarios presented in panel (a). These simulations also illustrate the effect of herd immunity, as the settings in the simulations do not allow any agent to be infected twice, the Rt decreases as a function of the number of infected agents that have become immune or died.”

**16) Line 675: Typo: counties ---> countries**
Thank you for pointing this out, we have corrected the typo.

**17) Figure 2 needs more explanation**
We have expanded the legend associated with this figure (now numbered as figure 1), to include a description of the dynamics characterising the number of tests performed and the number of pre-symptomatic and asymptomatic infections that escape the process of contact tracing and testing. The new text reads as follows (lines 695-711):

“Figure 1. Simulated evolution of the virus transmission over two regions. Illustration of two different simulations for the scenario 1 (i.e. identical seed) for the maps of southeast Italy (a) and the Midlands in UK (b). In the raster plots on the left, the dots represent the locations of the entire population of one hundred thousand agents. All simulations display the (failed) containment of the disease transmission relying only on contact tracing, testing, and isolation, under the conditions of medium incidence (25% daily increase in the number of infections), 20% contact tracing and testing efficacy, 20% of asymptomatic infections, deterministic transmission, and a distribution of travel cohorts of 40%, 30% and 30% for the short, medium and long travel range (respectively illustrated as black, blue and yellow squares overlaid on the maps). The right panels illustrate the oscillatory dynamic characterising the number of tests performed (magenta continuous line). The peaks in the number of tests performed never exhaust the daily testing capacity set for the simulations (horizontal dashed grey line). However, the process of contact tracing and testing constantly loses traces of the infection due to the low efficacy, determining the various dips in terms of the daily number of tests performed and consequently an increase in pre-symptomatic and asymptomatic infections (brown continuous line). Thus, this process failure in the attempt to find and isolate a sufficient number of infected agents to suppress or contain the reproduction of the infection is found irrespective of the testing capacity.”

**18) The reference style is not consistent for all the references.**
Thank for noticing this. We have now amended several entries also to update those articles that have meanwhile reached publication.

**Overall, this manuscript presents an interesting idea and the results validate the idea.**

Thank you for these detailed suggestions and the encouraging comments.
