# Supplementary material for: Containment of COVID-19: Simulating the impact of different policies and testing capacities for contact tracing, testing, and isolation
Source: PLoS One. 2021 Mar 31;16(3):e0247614. doi: 10.1371/journal.pone.0247614 (PMC8011755; doi:10.1371/journal.pone.0247614)
Supplement: Attachment — Submitted filename: Rebuttal_letter_secondroundrevision.docx [file pone.0247614.s002.docx]

Dear Dr. Yang,

Thank you, the additional editor and the reviewers for your comments. In this second revision we have now made every effort to address all remaining concerns, further clarifying the simulation procedures and settings.

We hope you will find the revised manuscript appropriate for publication in your journal.

Please see our point-by-point response to the reviewers below.

Sincerely,

Vincenzo Fiore, on behalf of all authors

Icahn School of Medicine at Mount Sinai – Department of Psychiatry

1 Gustave L. Levy Place, New York, NY 10029-5674

**Additional Editor Comments (if provided):**

**The authors have indeed improved this manuscript. I appreciate their efforts in adding a substantial amount of simulation results. Particularly, I’m glad to see the stochastic transmission being added. The addition of super spreader events is definitely a plus. Nevertheless, after reading the revised manuscript, I still found a lot of questions about the simulation procedure to be clarified, especially the contact tracing part.**

Thank you for your positive comments. Please find below our point by point response to your remaining concerns.

**1. Lines 107-108, “transmit the virus to up to 1, 8 or 25 healthy agents”, is it on each day or during the whole infectious period? My understanding from the methods is the former.**

You are correct: that value refers to the number of potential infections that a single infected agent can generate in one day. We have amended the text to clarify this point as follows (lines 106-108):

“Finally, three conditions of secondary infection cluster size were used to allow each infected agent to transmit the virus up to 1, 8 or 25 healthy agents, daily, depending on the condition.”

In the methods, lines 200-220 :

“We simulated one deterministic and two stochastic conditions (Figure 2e). In the deterministic condition, each day an infected agent was selected, it would transmit the virus to one single contact, randomly selected among those in the range of travel (Figure 1). […]

For instance, on average, under the larger-cluster stochastic condition, 4 out of 100 agents were assigned a secondary infection cluster size value of 8.75, meaning that each day they were selected they would transmit the virus to eight other agents with 100% probability, plus a ninth agent with 75% probability. Likewise, 10 agents were assigned a cluster size value of 0.17, meaning that each day they were selected they would transmit the virus to only one agent, with 17% probability.”

**2. Line 135, “we assume all symptomatic infections entered isolation the day they displayed symptoms.” Do you assume this for infected agents who were missed by contact tracing and testing? If so, make it clear in the discussion as a limitation that household transmission of non-hospitalized symptomatic infections is ignored.**

Infected agents that were found via contact tracing and testing were isolated starting from the following simulated day. Therefore, to be isolated at symptoms onset, the agents had to be missed by the process of contact tracing and testing. It is important to note that agents isolated at symptoms onset were still able to shed the virus in the three days prior the onset. We have amended the text to include the issue of household transmission in the limitations, as follows (lines 547-550):

“Concerning the third simplification and the immediate isolation of symptomatic infections, we acknowledge that this assumption implies that agents displaying symptoms would have immediate access to a test and would be able to either self-isolate or be isolated in a healthcare facility, thus avoiding even household transmission.”

**3. Line 145, what is “divided by four” for subclinical cases, mean of duration or the actual sampled duration? Why use Pearson distribution? There are different types of Pearson distributions. Which one is talked about here?**

To determine the several distribution setting, we used the Matlab built in function “pearsrnd()”, which creates random arrays from the Pearson system of distributions. The settings that we eventually used as an input for this function resulted in normal distributions: in the new amended manuscript we specify the type of distribution we are using (see the text below).

Concerning the setting controlling the number of days required to reach recovery, we used a two steps process: first we randomly assigned a value determining the number of days to reach recovery for all the agents in the population; second, we divided this number by four (rounding it) for those agents randomly selected to be part of the sub-clinical population, if infected [12,13].

We have amended the text to clarify both these points, as follows (lines 141-147):

“The number of days required to develop symptoms, if any (normal distribution: μ= 5.5; σ = 2; skewness = .6; Figure 2b), the number of days to reach full recovery, with a bimodal distribution due to the shorter time of recovery for the asymptomatic or paucisymptomatic [12,13] (μ= 21; σ = 5; skewness = 0, for all agents, with values assigned to the subclinical cases subsequently divided by four, so to reach recovery within 8 days in ~99% of the cases; Figure 2c), and the time spent in intensive care units (μ= 5; σ = 1.5; skewness = 0; Figure 2d) were also predetermined [39-41].”

**4. Line 146, is it necessary to simulate time in ICU? I don’t see how it impacts the results, as all symptomatic agents have already been isolated since symptoms onset.**

You are correct: this setting does not impact the results. Like other settings (e.g., mortality rate) it has been included to ease future development of the script and it is described in the text to ease the understanding of the output to be expected from the simulations. We have clarified this issue as follows (lines 147-150)

“To be noted that the settings controlling the ICU time or the recovery time for symptomatic agents did not affect the results, as all symptomatic agents were isolated at symptoms onset. These settings have been included only to ease future developments of the codebase.”

**5. The name “disease incidence” (15%, 25% and 35%) is very misleading. My understanding is these are percentages of infected agents that can actually transmit the virus, which is less than 1 because of nonpharmaceutical interventions other than contact tracing, isolation and quarantine. Why don’t you call it “relative transmission efficiency” or something like that?**

We acknowledge that the term “incidence” can be misleading and we have amended the text and figures to refer to “relative transmission efficiency”, as suggested.

**6. Lines 179-181, Rt also depends on the number of infections each infected agent can generate per day, or equivalently, the so called “size of infection cluster”. There are several misnomers or unnecessary new terms in this manuscript. “Size of secondary infections” would sound much better than “size of infection cluster”.**

We agree we use terms that have been borrowed from different fields. We have amended the text as you suggested, whilst keeping the reference to the cluster organization of the transmission. Through the text we now refer to the “secondary infection cluster size”.

We have decided to keep the reference to the infection clusters because the transmission routine in the simulations maybe described as generating a scale-free network pattern, under the two stochastic conditions of transmission. The use of the term cluster is meant to highlight the presence of a hub (the infected person) that can generate multiple contacts and therefore secondary infections. To the best of our knowledge, this use of the term is consistent with the use we have seen in literature, where it is associated with super-spreader events or household transmission, with one or few infected people generating multiple secondary infections (e.g. Adam et al 2020 Clustering and superspreading potential of SARS-CoV-2 infections in Hong Kong; Liu et al. 2020 Cluster infections play important roles in the rapid evolution of COVID-19 transmission: A systematic review).

**7. Line 185, I don’t see how the R0 (not Rt) values (3.05, 2.18 and 1.31) were calculated based on 35%, 25% and 15%. I guess you integrated over the Pearson distributions of the infectious period. Please give the exact formula. Also make it clear this is R0 of only a symptomatic case or a randomly selected infected agent (which could be subclinical).**

We have modified the text to refer to the R0 instead of the Rt. The calculation relies on a weighed sum to estimate the number of secondary infections generated by any agent during the entire time it remains infected and it is not isolated:

$$R_{0} = T_{e}(S_{t}+\sum_{{RH}_{t}=1}^{8} p_{{RH}_{t}} {RH}_{t})$$

Where T_e_ stands for the transmission efficiency (formerly, incidence), S_t_ stands for the time (expressed in days) prior to symptoms onset, RH_t_ represents the time (expressed in days) required to reach either recovery (in mild cases), or hospitalization (in severe cases) and $p_{{RH}_{t}}$ represents the probability assigned to each RH_t_ value.

We have amended the text as follows, as we also specified this estimation is estimated assuming an ideal condition in which contact tracing and testing is not in place and isolation only applies to severe cases, after hospitalization (lines 180-196):

“More precisely, in the absence of a testing and isolation strategy and without the isolation of symptomatic agents at symptoms onset [15], the R0 associated with each simulated condition of transmission efficiency was estimated using a weighted sum that considered: 1) the probability of each infected agent to be selected each day (i.e., the relative transmission efficiency setting); and 2) the number of days an infected agent could shed the virus (cf. Figure 2c), as follows:

$$R_{0} = T_{e}(S_{t}+\sum_{{RH}_{t}=1}^{8} p_{{RH}_{t}} {RH}_{t})$$

Where T_e_ stands for the transmission efficiency, S_t_ stands for the time (expressed in days) prior to symptoms onset, RH_t_ represents the time (expressed in days) required to reach either recovery (in mild cases), or hospitalization (in severe cases) and $p_{{RH}_{t}}$ represents the probability assigned to each RH_t_ value. We established conservative estimates assuming infected agents were allowed to transmit the virus for up to eleven days, i.e., the three days before symptoms onset, plus up to eight days due to either recovery, or hospitalization [12,13,41]. These settings led to R0 values of ~3.05, ~2.18, ~1.31, for the three settings of 35%, 25% and 15% incidence, respectively. These values are in keeping with current estimations for the COVID-19 [14,41,45-47] and allowed replicating the incremental dynamics in the number of infections reported by several countries (Figure 3).

**8. A lot of details about how contact tracing is simulated is still missing. Line 224, “a day-to-day pool of all agents displaying symptoms or found positive…”. What does “found positive” mean exactly? If by testing, when was the testing done? Per my understanding, your simulator does not distinguish between “contact tracing” and “tested positive”. I assume every active symptomatic agent, if not yet “tested and traced”, is eligible for testing and contact tracing. Whether this person will be tested and traced is random with the tracing and testing efficacy parameter as the probability.**

We acknowledge this description was not sufficiently detailed. What we meant here is that the pool of agents to be traced was composed of agents who had displayed symptoms as well as agents who had been “found” (i.e. tested positive) at any previous point in time via the process of contact tracing and testing. We have clarified this issue as follows (lines 233-249):

“Each day, the simulation of the contact tracing and testing process started after simulating the transmission of the disease. At day one of the simulated scenarios, the identity of the randomly selected .05% of the population that was infected was unknown to the system of contact tracing and testing. Thus, these agents were free to transmit the virus, starting three days before symptoms onset. However, the first day of symptoms onset, the agents that had become symptomatic were isolated and entered a pool that was updated on a day-to-day basis. This pool represented the starting point of the daily contact tracing and testing process. The agents listed in the pool were randomly selected one by one, removing them from future tracing pools and adding one positive test to the count of the day. Then, the agent under examination was used to trace the agent (if any) that had been the origin of her infection and the agents (if any) that she had infected at any prior time during the simulation. The process was repeated for all agents in the pool, until no agent remained to be examined or until exhaustion of the testing capacity (whichever came first). Those found positive via this contact tracing and testing process were isolated to prevent future transmission and entered the pool of traceable infected agents for the following day. As a consequence, all symptomatic agents eventually entered the pool of agents to be traced, by the time of symptoms onset. Conversely, asymptomatic and paucisymptomatic agents only entered the pool if found positive to a test, after being traced as the source or the target of another infected agent.”

**9. Line 226 “randomly selected one by one…”. What does “randomly selected” mean here, with what probability? Are all agents in this pool to be tested and traced? This comes back to the question of who were included in the pool. If people in this pool have not been tested and traced yet, then random selection makes for testing and tracing sense.**

The probability each infected agent in the pool has to be used for the contact tracing and testing process varies depending on the testing capacity and the number of agents to be traced. However, all infected agents in the pool are characterised by identical probability. For instance, if the pool consists of 5 infected agents, the process of contact tracing and testing will select one of these randomly (20% probability, each). The infections generated by this agent will be all analysed and either found positive or missed, depending on the contact tracing and testing efficacy. Then the process of contact tracing and testing will select a second infected agent from the pool, among the remaining 4 (25% probability, each). The process will repeat until exhaustion of the testing capacity or until the pool has been completely examined.

The amended text in the manuscript that clarified these details was reported in the answer to the previous comment (#8).

**10. Line 228, “agents (if any) that she had infected at any time…”. Do you mean the agents she or he infected both before and after the current time will be traced? On the current time step, how did you set agents that will be infected later to be traced?**

Any agent that was part of the test and tracing process was already isolated. Therefore it could not generate new infections in the future. To avoid the misunderstanding, we have modified the text to include a reference to the time, as follows (lines 241-243):

“Then, the agent under examination was used to trace the agent (if any) that had been the origin of her infection and the agents (if any) that she had infected at any prior time during the simulation.”

**11. Will asymptomatic/paucisymptomatic be tested and traced? If not, why? If yes, when did isolation start?**

Yes, they could be traced as contacts of other infected agents and become themselves part of the tracing and testing process. Once found positive, all agents would be considered isolated starting from the following day. We have amended the text as follows to clarify these points (lines 233-249):

“Each day, the simulation of the contact tracing and testing process started after simulating the transmission of the disease.

[…]

Those found positive via this contact tracing and testing process were isolated to prevent future transmission and entered the pool of traceable infected agents for the following day. As a consequence, all symptomatic agents eventually entered the pool of agents to be traced, by the time of symptoms onset. Conversely, asymptomatic and paucisymptomatic agents only entered the pool if found positive to a test, after being traced as the source or the target of another infected agent.”

**12. What does “tracing and testing” actually do to impact the simulation? Are traced and tested agents isolated immediately, regardless of symptom display? You mentioned all symptomatic agents will self-isolate upon symptoms onset. So the only difference contact tracing makes is to isolate infected contacts of identified agents (who have been tested positive and chose for tracing further contacts) before symptoms onset, is my understanding right?**

This is correct. The process of contact tracing and testing reduces the reproduction of the virus by allowing the isolation of asymptomatic and paucisymptomatic, who would not self-isolate, as well as by isolating pre-symptomatic agents, who would otherwise transmit the virus until symptoms onset. In the amended text we now report (lines 244-246)

“Those found positive via this contact tracing and testing process were isolated to prevent future transmission and entered the pool of traceable infected agents for the following day.”

**13. Lines 236-237, “but it would also leave undetected the entire chain of transmission associated with that missed agent”. I’m really confused here. Do you mean all contacts of the missed agent, and contacts of the contacts, will all be missed? If so, how do you make the pool mentioned in lines 224-225? On the other hand, for agents tested positive and chosen for contact tracing, how far will the contact tracing go, i.e., do you stop after his or her infected contacts were identified and isolated, or you move on to trace contacts of his or her infected contacts?**

What we meant here was that a missed infected agent would also result in missing the infections this agent had already generated (if any), which would be left free to shed the virus. For instance, in the case of super-spreader agents, missing an agent characterised by a secondary infection cluster size of 25 would potentially lead to neglect tens of infected agents.

We have amended the text as follows to clarify this point (lines 256-259):

“Importantly, a missed contact would not only result in leaving one infected agent free to transmit the disease, but it would also leave undetected the chain of transmission associated with that missed agent (i.e. the agents, if any, infected by the missed contact), until it could be traced via a new contact.”

Concerning the pool specifications, the amended text as reported in the response #8 has clarified this issue. Importantly, the new text also clarifies that the process of contact tracing and testing stops at the first iteration: newly found infections in day *t* are isolated and become part of the pool of agents to be traced at day *t+1*. The text has been amended as follows to clarify this point (lines 244-246):

“Those found positive via this contact tracing and testing process were isolated to prevent future transmission and entered the pool of traceable infected agents for the following day.”

**14. Lines 245-246, please give explicit calculations to show the equivalence, e.g., (1-25%) + 25% * (1-80%)=80%.**

We have amended the text to clarify the use of the percentages, as follows (lines 265-271)

“For instance, in a simulated scenario in which there are 100 infected contacts to trace and test, and a contact tracing and testing efficacy of 20%, 80 infected agents will avoid isolation on average. This may be due to a 25% efficacy in contact tracing jointly with 80% test sensitivity (25 contacts out of 100 are found, these 25 contacts are tested and 80% of these tests, 20, return positive), or 50% contact tracing efficacy with 40% test sensitivity (50 contacts out of 100 are found, and after being tested, 40% of the tests, 20, return positive). I.e., agents missed = traceable agents * (1- test efficacy * test sensitivity).”

**15. Lines 256-257, these 5000 infected and non-isolated agents are not necessarily going to be traced and tested on that day; therefore, isn’t adding 10-20 negative tests per active infected agent too many?**

We acknowledge this is an arbitrary setting. However we believe our setting is plausible in the sense that it uses a dynamic prevalence measure to adjust the ratio of positive and negative tests, irrespective of the number of tests performed or the daily testing capacity. Furthermore such simple mechanism of ratio with threshold complies with the objective of keeping the positivity rate below 5%, as by WHO requirement [7]. Thus, the estimations reached in the simulations provide plausible testing capacity values that allow to have a large number of negative tests per each positive one.

**16. Regarding testing policies aiding contact tracing described in lines 273-292, do you sample any agent, regardless of infection status and symptom status, or only sample infected but non-isolated agents (before symptoms onset and not traced yet)? If you mean the latter, it seems infeasible in reality. If the former, the underlying population seems too big, and it’s not clear how sampling is done. Do you use all the remaining test capacity for this sampling?**

The sampling policies used to aid contact tracing and testing targeted all healthy agents, as well as pre-symptomatic, paucisymptomatic and asymptomatic infections; in other words, all the agents who were not already isolated due to a known infection could be selected for a random test. The point of these policies is not to find all infections, but to feed the contact tracing and testing process with a few missed chains of transmissions, preventing the process from being idle. The improvement in a comparison with the use of the contact tracing and testing policy alone is significant across all conditions and scenarios (cf. Figure 5).

Concerning the testing capacity for the sampling policies, these were developed to use only the test capacity left unused by the contact tracing and testing process, so the number of actual sampling tests performed daily varies.

We have amended the text as follows to clarify these points (lines 313-319):

“The potential target of these sampling testing policies were all healthy agents, as well as pre-symptomatic, paucisymptomatic and asymptomatic infections; in other words, all the agents who were not already isolated due to a known infection could be selected for a random test. The number of agents tested in these aiding policies was determined daily, so as to exhaust the testing capacity left unused by the process of contact tracing and testing. The agents found positive would then be isolated (i.e. they could not contribute to the future transmission of the virus) and would be included in the pool of agents to be traced, starting from the subsequent simulated day”

Finally, concerning the sampling policies used, we report in the methods that these are “variations of weighted sampling within a small ‘cell’, replicating the dimension of the small ‘sector’ used for the travel behaviour (a square with a side of 100 pixels, cf. Figure 1).” (lines 299-301). Since the weighted sampling uses the simplified behaviour of the agents as a measure to establish the weights, we report in the Discussion that these policies are “meant to illustrate that general information about population distribution and range of movement can ideally be used to improve the containment of the disease.” (lines 517-518)

**17. In line 376, you mentioned “mean number of infected agents recorded AT day 60”. In line 389, you stated “the number of infections recorded BY the last simulated day”. Which is correct?**

Thank you for pointing this out: it was supposed to be “at” in both cases. We have amended the text to correct the mistake.

**Reviewer #1: (No Response)**

**Reviewer #2: Thank you so much for addressing my previous concerns. I believe the current version is acceptable for publication after minor revisions of the following comments.**

Thank you for your positive comments.

**1) Lines 344-345: Please correct the order of the numbers related to medium or high incidence to be compatible with the text.**

Thank you for pointing this out, we have amended the text as suggested.

**2) Lines 450-451: Rewrite "we set the testing capacity so that it would not be exhausted during most of the simulated time(i.e., more than 50 simulated days".**

Thank you for this suggestion. We have amended the text as follows (lines 479-480):

“we set the testing capacity so that it would not be exhausted for at least 50 of the total 60 simulated days.”
